# Service in Hospital Wards

**Published:** 1913-01-25

**Authors:** Laura Lawson Johnston

**Affiliations:** 29 Portman Square, W.


*To the Editor of* The Hospital.

### Service in Hospital Wards.

*To the Editor of* The Hospital.

Sir,— As I am much interested in hospitals, being a subscriber in some way or another to a good many, may I be excused for asking a question concerning them in your columns?

I heard first hand a day or two ago of a late patient in one of the large London hospitals complaining that there had been no Church service or any form of worship on Sundays in the ward. Is this the case in many hospitals? If so, it seems to me a pity the patients should not be reminded in some small way of the Sabbath, even if it is only by a little hymn-singing or one or two prayers which, I feel sure, would gladden those who were suffering or had just come through a bad time.—Yours faithfully,

Laura Lawson Johnston.

29 Portman Square, W.,
January 21, 1913.

[We shall be glad if Mrs. Lawson Johnston will send us the name of the hospital to which she refers, as it is probably unique, if the facts turn out to be as stated. In all well administered hospitals there is a chaplain, and the sister in each ward holds a little service, with hymn-singing usually, on most evenings in the week.—Ed. The Hospital.]

